# Cultural Variation in the Use of Overimitation by the Aka and Ngandu of the Congo Basin

**DOI:** 10.1371/journal.pone.0120180

**Published:** 2015-03-27

**Authors:** Richard E. W. Berl, Barry S. Hewlett

**Affiliations:** 1 School of Biological Sciences, Washington State University, Pullman, Washington, United States of America; 2 Department of Anthropology, Washington State University Vancouver, Vancouver, Washington, United States of America; University of Edinburgh, School of Philosophy, UNITED STATES

## Abstract

Studies in Western cultures have observed that both children and adults tend to overimitate, copying causally irrelevant actions in the presence of clear causal information. Investigation of this feature in non-Western groups has found little difference cross-culturally in the frequency or manner with which individuals overimitate. However, each of the non-Western populations studied thus far has a history of close interaction with Western cultures, such that they are now far removed from life in a hunter-gatherer or other small-scale culture. To investigate overimitation in a context of limited Western cultural influences, we conducted a study with the Aka hunter-gatherers and neighboring Ngandu horticulturalists of the Congo Basin rainforest in the southern Central African Republic. Aka children, Ngandu children, and Aka adults were presented with a reward retrieval task similar to those performed in previous studies, involving a demonstrated sequence of causally relevant and irrelevant actions. Aka children were found not to overimitate as expected, instead displaying one of the lowest rates of overimitation seen under similar conditions. Aka children copied fewer irrelevant actions than Aka adults, used a lower proportion of irrelevant actions than Ngandu children and Aka adults, and had less copying fidelity than Aka adults. Measures from Ngandu children were intermediate between the two Aka groups. Of the participants that succeeded in retrieving the reward, 60% of Aka children used emulation rather than imitation, compared to 15% of Ngandu children, 11% of Aka adults, and 0% of Western children of similar age. From these results, we conclude that cross-cultural variation exists in the use of overimitation during childhood. Further study is needed under a more diverse representation of cultural and socioeconomic groups in order to investigate the cognitive underpinnings of overimitation and its possible influences on social learning and the biological and cultural evolution of our species.

## Introduction

Human behavior is the result of a complex coevolutionary history written by the network of interactions between our biology, our culture, and our environment [1, 2]. From this rich history has arisen a great deal of behavioral variability both within and between human groups [3]. That many aspects of behavior in humans and other animal species are not innate but socially learned from parents, other adults, or peers means that the acquisition of behavior is highly contingent upon an individual’s sociocultural environment. Further, enculturation begins in infancy and early childhood [4–7], possibly even in utero [8], suggesting that there is no such thing as a “culture-less child.” Hence, it is unlikely that any socially learned behavior or the cognitive mechanisms underlying its transmission and acquisition are ever unaffected by an individual’s particular culture and ontogeny.

These concerns underscore the importance of cross-cultural comparative research in psychological, anthropological, and biological lines of inquiry that address human behavior and cognition, particularly those concentrating on social learning, cultural transmission, and associated mechanisms that fall under the study of cultural evolution [9–13]. This need is especially crucial given that often the primary focus of these studies is to search for core mechanisms and processes that are universal to modern humans. It is surprising, then, that very few studies have included non-Western samples in their analyses. Undergraduate university students enrolled in psychology courses constitute the sole sample for 67% of psychological studies based in the United States and 80% of studies elsewhere [14]. Only 18% of samples from leading psychology journals in the mid-2000’s were from non-English speaking countries, with the majority of those non-English samples coming from highly industrialized countries in Western Europe. This has left 95% of the research effort concentrated on a heavily biased subsample drawn from just 12% of the world’s population [14]. It is thus inappropriate to attempt to generalize findings from this restricted sample of Western people to any non-Western group—or, indeed, to other Western cultures or to sub-cultures with different socioeconomic conditions—without additional sampling sufficient to explore the breadth of cross-cultural variation [3].

Much recent interdisciplinary effort in social learning research has been focused on by what means and under which conditions humans and other animal species employ a number of possible social learning mechanisms and strategies [15–18], with the most basic of these being imitation and emulation. Following Whiten et al. [19], we refer here to the broad definitions of imitation and emulation, with imitation as a focus by the observer on the reproduction of the form of modeled actions rather than the result or goal of those actions and, conversely, with emulation as a focus on reaching the modeled outcome rather than on the details of the actions that accomplish that outcome. The phenomenon of “overimitation”—though the term was coined by Lyons et al. [20]—was first observed by Horner and Whiten [21] in a study investigating whether children (from two nursery schools in the United Kingdom) and captive chimpanzees could switch between imitative and emulative mechanisms given the availability of visual information about causal relationships. In the experiment, a model demonstrated a series of actions by which a reward could be retrieved from an artificial apparatus. Some of the demonstrated actions were causally relevant and necessary to obtain the end reward, but others were shown to be irrelevant and could be omitted by using emulation, thereby increasing the efficiency of the procedure and reaching the reward more quickly. They found that the chimpanzees did so but that the children—even in the presence of visual information about the causality of their actions—continued to imitate the irrelevant demonstrated actions rather than switching to an emulative approach. **Overimitation** is thus defined here as *the high-fidelity copying of causally irrelevant actions in the presence of clear causal information*.

Subsequent studies have observed overimitation in Western children under a variety of experimental conditions. It appears to develop between the ages of 18 months and three years [22–24], to increase through ages three to five [20, 21, 25–27], and is observed at even higher rates in adults [28, 29]. Whether it persists through adolescence or re-emerges in early adulthood is presently unknown due to the lack of studies in this developmental period. Curiously, overimitation appears to be largely absent in children up to at least 18 months [23, 30, 31] and different social or contextual cues can trigger selective copying at the same ages in which overimitation is observed (for a review, see [32]). Many of these studies have attempted to tie overimitation into current theory on the cultural and biological evolution of our species, as a non-selective copying strategy has the potential to facilitate rapid adoption of the vast amount of causally opaque cultural knowledge available during childhood [27, 28, 33–40]. Nielsen and colleagues have suggested that overimitation is a “universal human trait” ([27] (p. 729), [36] (p. 156), [39] (p. 171)).

To date, there have been only two studies of overimitation conducted with non-Western groups. Nielsen and Tomaselli [27] investigated overimitation in the !Xóõ, ‡Khomani, !Xun, and Khwe San peoples of Botswana (!Xóõ) and South Africa (‡Khomani, !Xun, and Khwe), while Nielsen et al. [40] extended this research to two groups of Aboriginal Australians, the Yanyuwa and the Garrwa in the Northern Territory, and performed additional work with the San groups from the previous study. Both studies found little difference between the indigenous populations and a group of Western children in Brisbane, Australia in the frequency or manner with which children overimitate.

However, there is good reason to believe that the San and Aboriginal Australians involved in these studies are no longer actively engaged in a way of life typical of hunter-gatherers or other small-scale cultures. Robins [41] describes the present-day ‡Khomani San as “a group of superexploited and hypermarginalized ex-farm workers.” The San people are a loose affiliation of indigenous groups that have had continuous exposure to Western ideas, values, and technology for many decades, and this life beside and within Western society has fundamentally altered their respective cultures [41–43]. In all cases, the San have been made to adapt to political, social, economic, and religious pressures exerted by Western influences (see **S1 Appendix**). Aboriginal Australian groups have been subjected to similarly intense pressures throughout their history of contact and conflict with Western cultures [44].

In considering these two non-Western studies and a third incorporating a sample of children from Colombo, the urban, industrialized de facto capital of Sri Lanka [38], each additional cultural group provides valuable data toward the examination of overimitation and other social learning processes and broadens cultural and socioeconomic representation in this line of research. With this being said, we agree with the assessment of Whiten [45] that it is inappropriate at present to claim human universality of a trait that has only been observed in such a narrow span of the world’s cultures. Speculation on the significance of overimitation in the evolution of human cumulative culture has been similarly overextended beyond the limits of our knowledge of its underlying causal mechanisms.

In an effort to investigate overimitation in a context with limited Western cultural influences, we conducted a study with the Aka hunter-gatherers and the neighboring Ngandu horticulturalists of the Congo Basin rainforest in the southern Central African Republic. The Aka (also referred to as the Biaka or Bayaka) are a group of foragers that express the set of “pygmy” phenotypic traits presumed to be adaptive for life in the tropical rainforest [46–51]. About 40,000 Aka inhabit the equatorial rainforests of the northern Republic of the Congo and southern Central African Republic, with about 2,000 living in and around the study area. The Aka live in highly mobile groups of 25–35 people, typically made up of families related by kinship and marriage, which move camp several times per year. They actively rely upon a wide variety of hunting and gathering techniques for their day-to-day subsistence.

The Ngandu are a group of Bantu-speaking farmers that live in villages of 50–200 individuals and cultivate manioc, corn, plantains, and peanuts. They exchange some of their domesticated crops for meat and other forest products from the Aka. Women plant, maintain, and harvest the fields and provide the majority of dietary calories while men fish, hunt, and trade. About 15,000 Ngandu live mostly within the Central African Republic, with about 4,000 living in and around the study area.

The Aka in this study have complex economic, ritual, and kinship relationships with the Ngandu [4, 52] similar to those seen in many central African hunter-gatherer groups associated with farmers [53, 54]. The Aka and Ngandu speak closely-related Bantu languages (Ethnologue identifiers *axk* and *ngd*, respectively) [55], though a portion of the Aka language in certain domains is shared with other foragers in the Congo Basin [56]. The Aka are in close contact with the Ngandu but, because the area as a whole is relatively isolated due to the lack of local infrastructure, the Aka themselves have limited exposure to Western influences, do not live under socio-economic poverty, receive no government assistance or subsidy, and maintain their foraging culture and social norms with relatively few outside pressures ([46, 50, 53] and see **S1 Appendix**).

Given that overimitation has been observed in children from every culture examined thus far, it was predicted that Aka and Ngandu children also would overimitate. However, few studies have focused on children from cultures that are not Western, educated, industrialized, rich, and democratic (“WEIRD” [3]). This makes definitive predictions difficult, as there are a number of foundational differences between WEIRD cultures and small-scale non-Western ones that structure their perceptions and values and lead to variation in behavior ([3] and see **S2 Appendix** on the Aka and Ngandu). Additionally, though the Ngandu are largely non-Western and have daily contact with the Aka, the Ngandu receive formal education through schooling (see **S1 Appendix**) and emphasize respect for elders and those of higher status (see **S2 Appendix**), which could be expected to generate differences between Aka children and Ngandu children in their approaches to social learning.

## Methods

### Ethics Statement

All participants provided verbal informed consent to take part in the study after having been read and acknowledging that they understood an approved consent script. Adults provided consent to the researcher in the presence of a witness, as did participating children and their parents. Written consent could not be obtained because most of the participants were not literate. Research protocols, the use of verbal consent, and verbal consent scripts were approved by the Washington State University Institutional Review Board (protocol #12344).

### Participants

Three groups were recruited for the study, consisting of 28 Aka children, 29 Ngandu children, and 14 Aka adults. Ages ranged from 4 to 7 years in Aka children (*M* = 5.4, *SD* = 1.2), from 4 to 7 years in Ngandu children (*M* = 5.3, *SD* = 0.9) and from 20 to 38 years in Aka adults (*M* = 28.4, *SD* = 6.0). Sex representation was equal or approximately equal in all groups (Aka children: 14M, 14F; Ngandu children: 14M, 15F; Aka adults: 7M, 7F). Ngandu adults could not be sampled due to field research constraints and ongoing political unrest in the Central African Republic.

### Apparatus

**Fig 1:**
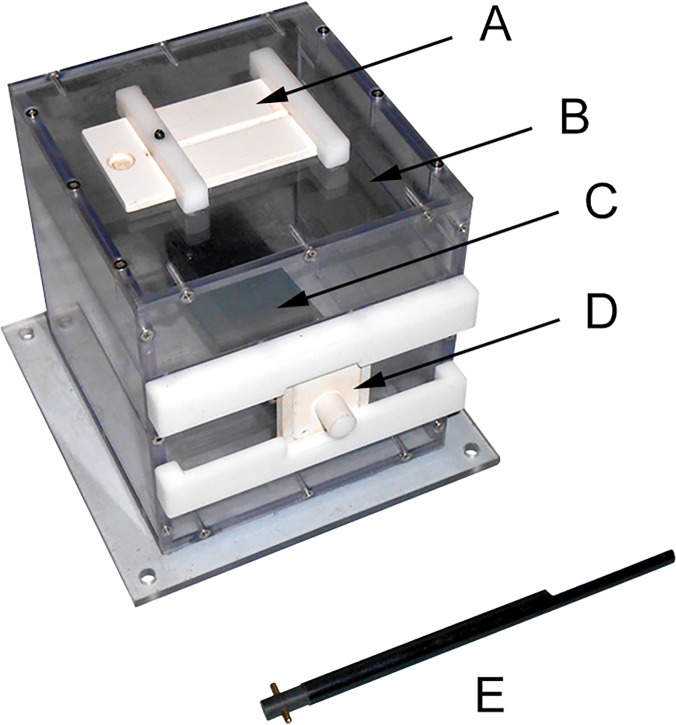
Experimental apparatus and tool. Used for all participants and tests in this study. Relevant features include: (A) sliding top door; (B) transparent barrier (below the plane of the top side of the apparatus); (C) opaque reward chamber; (D) sliding/lifting front door; and (E) manipulating tool (shown to scale with apparatus). See Methods for details of construction and use.

The study apparatus was a transparent polycarbonate box (see **Fig. 1**), measuring roughly 15 cm x 15 cm x 15 cm and similar to those used by Horner and Whiten [21], McGuigan et al. [26], McGuigan and Whiten [22], and McGuigan et al. [28], differing only in the top door mechanism. Two holes (each 2 cm x 2 cm) allowed entry into the box, one on the top side and one on the front side. The front hole was covered by a door with a small knob and allowed entry by sliding the door to either side or by lifting it upward and outward. The top hole was covered by a sliding door with an open notch and could be slid only to the left. A 22-cm-long aluminum tool with two short knobs protruding from one end and a long flat section on the opposite end was used to interact with the box and retrieve the reward—a small piece of bubble gum—which was stored in an opaque black rectangular prism connected to the inside of the front hole. The top hole led to a chamber with a transparent barrier separating it from the rest of the box and the reward. Thus, demonstrated actions performed on the top or sides of the box were causally irrelevant to obtaining the reward. Opening the front door and obtaining the reward were judged to be causally relevant.

### Design

Participants in each group were randomly assigned to receive a demonstration or to receive no demonstration, such that roughly one third of the available participants in each group were assigned to the no demonstration condition (Aka children: *N* = 9; Ngandu children: *N* = 9; Aka adults: *N* = 4). In the demonstration condition, participants each observed an ingroup adult male model as he performed a sequence of actions on the box which resulted in the retrieval of the reward. Participants in the no demonstration condition had the box and tool placed in front of them without any actions demonstrated by the model. The Aka and Ngandu models were both members of the local community, in their late thirties, and had been prior research assistants for the second author. They were known to the participants but not familiar to them and were not close relatives of any participant in the study. Neither held a position of particular status within the community.

### Procedure and Coding

Tests were conducted in locations familiar to the participants. Aka children and adults were tested in a camp house or on a nearby forest trail and Ngandu children were tested in a village home. The participant sat next to the model, with the apparatus and tool placed in front of and midway between the model and participant. In the demonstration condition, the model said “Watch me,” and demonstrated the complete sequence three times, reloading the box out of view of the participant after each demonstration. He then placed the box and tool in front of the participant, said “Your turn,” and did not interact with the participant for the remainder of the test.

The demonstrated sequence consisted of 6 actions, with the first 4 being causally irrelevant and the final 2 being causally relevant to the goal of obtaining the reward: tap the right side of the box (“TR,” *irrelevant*), tap the left side of the box (“TL,” *irrelevant*), slide the top door (“ST,” *irrelevant*), tap the barrier inside the top door (“TT,” *irrelevant*), slide the front door (“SF,” *relevant*), and retrieve the reward (“RR,” *relevant*). Thus, the complete demonstrated sequence in order was: TR, TL, ST, TT, SF, RR. Participants were given up to one minute to interact with the apparatus and, if they did so, two minutes to retrieve the reward.

In the no demonstration condition, after the box and tool were placed in front of and between the model and participant, the model said, “Can you find the treat?” and did not interact with the box while feigning disinterest for the rest of the test. Participants in the no demonstration condition were prompted with this phrase in order to provide context for the task as a puzzle, which would otherwise be absent given that the highly artificial nature of the experimental apparatus was incongruent with that of a naturalistic foraging problem. Circumstances were otherwise identical to the demonstration condition.

All tests were recorded on video for coding and review. Trials were coded in randomized order. Coding of tests included only the six demonstrated actions; other actions were noted for the purpose of determining copying fidelity but were not coded as relevant or irrelevant due to their inherent ambiguity of purpose.

## Analysis

From the coded data—in addition to the number of irrelevant actions performed—three other measures were calculated for each test. First, an “irrelevant imitation score” [21, 22, 26, 28, 57] was generated by dividing the number of tool or hand insertions into the top irrelevant hole (TT) by the total number of insertions into both the top irrelevant and front relevant holes (TT + RR). This measurement ranges from 0, meaning that all insertions were relevant, to 1, meaning that all insertions were irrelevant. Trials where no insertions took place and, by consequence, the reward was never retrieved could not be given an irrelevant imitation score.

Second, an “irrelevancy quotient” was determined by dividing the total number of irrelevant actions (TR + TL + ST + TT) by the total number of irrelevant and relevant actions (TR + TL + ST + TT + SF + RR), and thus is simply the proportion of actions performed by the participant that were irrelevant. Participants that did not engage with the task and therefore did not perform any actions on the box were not given an irrelevancy quotient. This measure was calculated in order to represent the full proportion of irrelevant actions performed by the participant rather than limiting observations to insertions into either hole on the box, which was originally done due to methodological constraints [21]

A proportional measure such as the irrelevant imitation score or irrelevancy quotient is necessary because strictly examining the number of irrelevant actions does not account for variance in the total number of actions performed. An equal number of irrelevant actions could be obtained from a situation in which a few irrelevant actions were performed along with a few relevant ones, or from one in which a great number of actions were performed and a small proportion of them were irrelevant. The irrelevant imitation score and irrelevancy quotient take this issue into account by providing measures of the relative irrelevancy of the full complement of actions performed.

The irrelevancy quotient also allows for comparison with studies that may not have one primary irrelevant action suitable to be paired with one relevant reward retrieval action, as in the irrelevant imitation score. For a more rigorous comparison between studies, an adjustment should be made to standardize irrelevancy quotients by the proportion of irrelevant actions in the demonstration—which in this case is 4 out of 6, or two thirds—but obtaining the raw data and calculating this for each presently available study is beyond the scope of this paper.

Third, a “fidelity quotient” was calculated by first determining a fidelity score, defined as the longest string of actions performed in the same order as the demonstration. For example, a trial consisting of the actions TR, TL, ST, SF, RR would have a fidelity score of 3, while the actions TR, TL, SF, RR would have a fidelity score of 2 for the particular demonstrated sequence of this study (TR, TL, ST, TT, SF, RR; as above). Higher fidelity scores indicate greater attention to the imitation of sequential structure, which is useful in distinguishing imitation from other transmission mechanisms [58, 59]. The fidelity score is then divided by the maximum possible fidelity score, i.e. the length of the demonstrated sequence (6 for this study), to obtain the fidelity quotient. The calculation of a fidelity quotient yields an easily-determined quantitative measure of the level of copying fidelity given a short sequence of demonstrated actions. For experiments in which the demonstrated sequence contains irrelevant actions, the fidelity quotient complements the irrelevancy quotient in assessing overimitation, since overimitation (as defined for this study) requires both the reproduction of irrelevant actions and high copying fidelity. As with the irrelevancy quotient, a fidelity score or quotient could not be assigned for trials in which no actions were performed on the box.

The social learning mechanism employed by each participant was determined based upon the actions exhibited following the demonstration. As in previous studies, the use of one or more irrelevant actions that matched those demonstrated constituted imitation, whereas the exclusion of these irrelevant actions qualified as emulation [21, 22, 26]. Participants that ignored the apparatus or did not complete the test could not be assigned a social learning mechanism unless an irrelevant action matching the demonstration had been used.

As an additional measure of interest, the duration of each test was timed from the first action performed on the box by the participant (after any demonstrations) to the time when the reward was successfully retrieved. No duration was recorded for participants that did not participate or failed to retrieve the reward. This measure was recorded, as in McGuigan et al. [26] and McGuigan and Whiten [22], to investigate if either social learning mechanism led to a higher task efficiency.

An independent observer unfamiliar with the experimental hypotheses coded 15 trials, selected at random from the total 58 in which participants engaged with the test, representing 26% of the total sample. The observer and experimenter agreed on the specific coding of observed actions to a high degree (81%, Cohen’s κ = .778).

Additional details on the methods and statistical tests performed can be found in **S3 Appendix**.

## Results

### Control

Univariate factorial ANOVA tests were used to examine the main effects and interactions of demonstration condition (demonstration or no demonstration), group (Aka children, Ngandu children, or Aka adults), and sex (male or female) on each dependent variable: the number of irrelevant actions, irrelevant imitation scores, irrelevancy quotients, and fidelity quotients.

**Fig 2:**
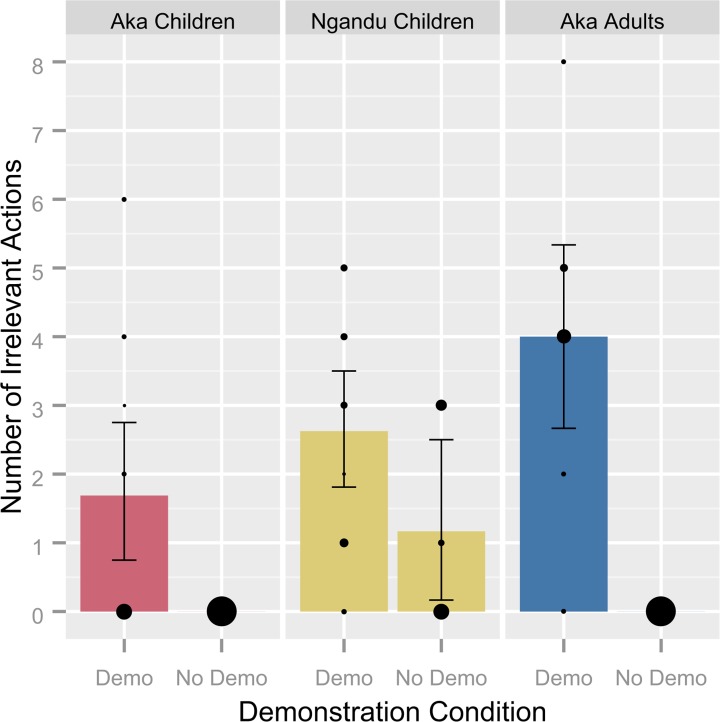
Number of irrelevant actions by group and demonstration condition. Bars indicate sample means and 95% confidence intervals, computed through basic nonparametric bootstrapping. Bubbles show the distribution of values within each sample; the size of each bubble represents the relative proportion at that value.

Participants in the demonstration condition were found to exhibit a higher number of irrelevant actions than participants in the no demonstration condition (F[1, 45] = 4.138, p = .048, ƞ_p_^2^ = .084, 95% CI [.275, 2.387]; see **Fig. 2**). Multiple comparisons were unable to make distinctions between conditions within groups due to the low number of participants in the no demonstration condition that engaged with the experiment (Aka children: *N* = 4; Ngandu children: *N* = 6; Aka adults: *N* = 3). Irrelevant imitation scores and irrelevancy quotients did not vary by condition (95% CIs [−.120, .233] and [-.411, .110], respectively) while fidelity quotients were lower in the no demonstration condition (F[1, 51] = 18.822, p << .001, ƞ_p_^2^ = .270, 95% CI [.198, .539]). Sex was not a significant predictor for any measure and no significant interaction effects were found.

For methodological comparison, Welch’s t-tests and Pearson’s chi-squared tests were conducted as in Nielsen and Tomaselli [27] and Lyons et al. [20], respectively, on the number of irrelevant actions, irrelevant imitation scores, irrelevancy quotients, and fidelity quotients collapsed across groups. Results of these tests agreed qualitatively with findings from the ANOVA F-tests for all t-tests (number: t[19.538] = 3.226, p = .004; fidelity: t[52.932] = 6.258, p << .001) and chi-squared tests (fidelity: *Χ* ^2^ [1, *N* = 56] = 5.628, p = .018). The chi-squared test of the number of irrelevant actions became equivalent to that of the irrelevancy quotient due to both being converted to count data.

Subsequent analyses below pertain solely to participants within the demonstration condition (Aka children: *N* = 19; Ngandu children: *N* = 20; Aka adults: *N* = 10). Full ANOVA tables for all analyses, including confidence intervals and effect sizes for significant and nonsignificant effects, are available in the supporting information (see **S1 Table**; **S2 Table**; **S3 Table**).

### Reproduction of Irrelevant Actions

Following the control analyses on demonstration condition, univariate factorial ANOVA tests were used to examine the main effects and interactions of group (Aka children, Ngandu children, or Aka adults) and sex (male or female) on each measure of irrelevancy: the number of irrelevant actions, irrelevant imitation scores, and irrelevancy quotients. ANCOVA was used to test for differences by group (Aka children or Ngandu children) due to the effects of participant age (continuous) on these same measures, excluding Aka adults to avoid confounding group with age.

The number of irrelevant actions performed by each participant was found to vary by group (F[2, 37] = 3.706, p = .034, ƞ_p_^2^ = .167; see **Fig. 2**), with post-hoc Tukey’s HSD confirming that Aka children performed fewer irrelevant actions than Aka adults (p = .026, 95% CI [.243, 4.382]), while other groupwise comparisons were nonsignificant (Aka children and Ngandu children: p = .402, 95% CI [-.819, 2.694]; Ngandu children and Aka adults: p = .249, 95% CI [-.695, 3.445]). There was no difference by sex in the number of irrelevant actions performed and no difference was attributable to the age of the participant within or between the two groups of children. All interaction terms were found to be nonsignificant.

**Fig 3:**
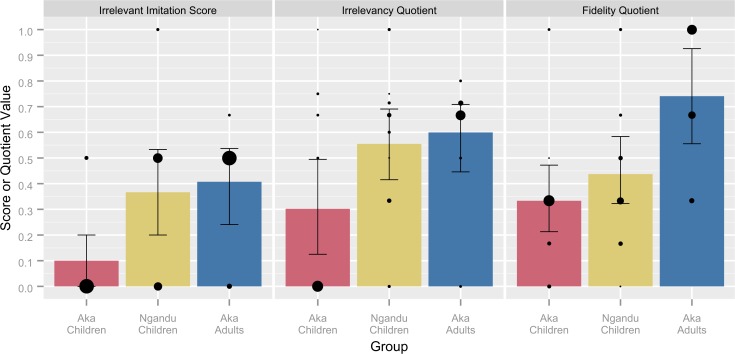
Measures of irrelevancy and fidelity by group. Irrelevant imitation scores and irrelevancy quotients represent proportions of irrelevancy and fidelity quotients represent copying fidelity (see Analysis for descriptions). Bars indicate sample means and 95% confidence intervals, computed through basic nonparametric bootstrapping. Bubbles show the distribution of values within each sample; the size of each bubble represents the relative proportion at that value.

Irrelevant imitation scores differed between groups (F[2, 35] = 4.501, p = .018, ƞ_p_^2^ = .205; see **Fig. 3**), with Aka children having lower scores than both Ngandu children (Tukey’s HSD, p = 0.034, 95% CI [.017, .516]) and Aka adults (Tukey’s HSD, p = 0.035, 95% CI [.019, .596]), but with no difference between Ngandu children and Aka adults (Tukey’s HSD, p = .936, 95% CI [−.248, .329]). No differences were found by participant sex or age and all interaction terms were found to be nonsignificant.

Irrelevancy quotients were also found to differ between groups (F[2, 37] = 3.332, p = .036, ƞ_p_^2^ = .153; see **Fig. 3**), but post-hoc tests were not conclusive (Tukey’s HSD, Aka children and Ngandu children: p = .077, 95% CI [−.022, .528]; Aka children and Aka adults: p = .078, 95% CI [−.027, .622]; Ngandu children and Aka adults: p = .940, 95% CI [−.280, 0.369]). No differences were found by participant sex or age and all interaction terms were found to be nonsignificant.

### Copying Fidelity

To then examine copying fidelity, a univariate between-groups factorial ANOVA was used to examine the main effects and interactions of group (Aka children, Ngandu children, or Aka adults) and sex (male or female) on fidelity quotients. ANCOVA was used to test for differences by group (Aka children or Ngandu children) due to the effects of participant age (continuous) on fidelity quotients, again excluding Aka adults.

Fidelity quotients varied by group (F[2, 39] = 6.268, p = .004, ƞ_p_^2^ = .243; see **Fig. 3**), such that Aka children had lower scores than Aka adults (Tukey’s HSD, p = .003, 95% CI [.124, .690]) and Ngandu children had lower scores than Aka adults (Tukey’s HSD, p = .038, 95% CI [.014, .592]) while there was no difference seen between Aka children and Ngandu children (Tukey’s HSD, p = .541, 95% CI [−.134, .342]). No differences were found by participant sex or age and all interaction terms were found to be nonsignificant.

### Social Learning Mechanism

**Fig 4:**
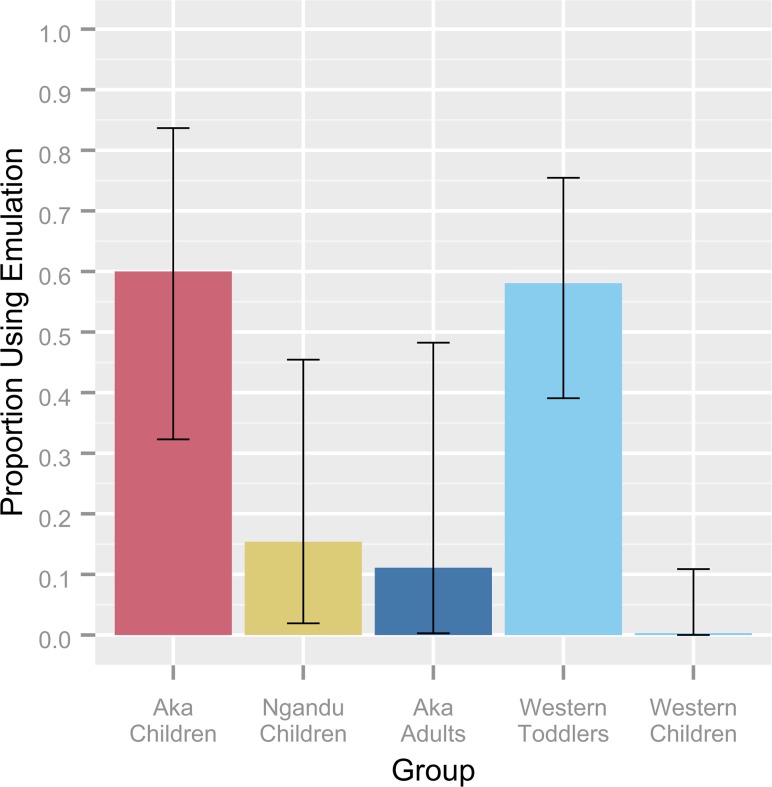
Use of emulation by group. Bars indicate the proportion of those participants that succeeded at obtaining the reward that did so using an emulative social learning mechanism, wherein the copying of one or more irrelevant actions matching the demonstration constituted imitation and the copying of zero matching irrelevant actions constituted emulation (see Introduction and Analysis). Error bars indicate 95% confidence intervals, computed using the Clopper-Pearson (exact) method. Frequencies of the use of emulation by Western toddlers and children from the United Kingdom are included for comparison. Data for these groups are from overimitation studies by McGuigan et al. [26] and McGuigan and Whiten [22] and are summarized in Table 1. The two age classes within each Western group are collapsed here for clarity.

Only participants that succeeded in obtaining the reward (Aka children: *N* = 15; Ngandu children: *N* = 13; Aka adults: *N* = 10) were analyzed in terms of the social learning mechanism used. The proportion of individuals that succeeded by use of emulation rather than imitation differed by group (Fisher’s Exact, p = .017; see **Fig. 4**), with Aka children using emulation more often than Ngandu children (Fisher’s Exact, p = .024) and more often than Aka adults (Fisher’s Exact, p = .033) with no difference seen between Ngandu children and Aka adults (Fisher’s Exact, p = 1.000). The use of emulation was a significant predictor of the number of irrelevant actions (*β* = −3.720, t(35) = −7.435, p << .001), of irrelevant imitation scores (*β* = −.347, t(35) = −4.867, p << .001), of irrelevancy quotients (*β* = −.614, t(35) = −15.870, p << .001), and of fidelity quotients (*β* = −.377, t(35) = −4.268, p < .001). A significant proportion of the variance in the number of irrelevant actions (r^2^ = .601, F(1, 35) = 55.28, p << .001), irrelevant imitation scores (r^2^ = .387, F(1, 35) = 23.68, p << .001), irrelevancy quotients (r^2^ = .874, F(1, 35) = 251.7, p << .001), and fidelity quotients (r^2^ = .324, F(1, 35) = 18.22, p < .001) was explained by the use of emulation. No differences were found by participant sex or age and all interaction terms were found to be nonsignificant.

These results were compared with data from overimitation studies by McGuigan et al. [26] and McGuigan and Whiten [22], which report the social learning mechanisms used by toddlers and children from nursery schools and parent-toddler groups in the United Kingdom (see **Fig. 4**; **Table 1**). Aka children displayed levels of emulation comparable with 23-month-old and 30-month-old Western toddlers, but emulated at much higher frequencies than 42-month-old (Fisher’s Exact, p = .003) and 66-month-old Western children (Fisher’s Exact, p = .003). The frequencies of emulation seen in Ngandu children and Aka adults could not be distinguished from any of these Western groups. At a mean age of 5.4 years, or roughly 65 months, the Aka children that succeeded at the task were much older than the comparably emulating toddler groups (see **Table 1**). Though a direct comparison cannot be made between the Aka and Ngandu participants and the San studied by Nielsen and colleagues [27, 40], the San participants were of ages that ranged both younger and older than the Aka children studied here and had high frequencies of overimitation at all ages tested ([27]: *M* = 5.5, *SD* = 3.1, range = 2–13; [40]: *M* = not reported, *SD* = not reported, range = 3–6).

**Table 1:** Comparison of the use of emulation by Aka children, Aka adults, and Ngandu children with Western toddlers and children.

Group	Comparison Group	Use of Emulation			P	Age in Years		
		Proportion	Lower 95% CI	Upper 95% CI		M	SD	Range
Aka Children	—	0.600	0.323	0.837	—	5.4	1.2	4.0–7.0
	23-m/o Western Toddlers	0.667	0.384	0.882	1.000	1.9	0.3	1.5–2.0
	30-m/o Western Toddlers	0.500	0.247	0.753	1.000	2.5	0.3	2.2–2.9
	42-m/o Western Children	0.000	0.000	0.206	**.003**	3.6	0.3	3.2–4.0
	60-m/o Western Children	0.000	0.000	0.206	**.003**	5.4	0.3	5.0–5.8
Ngandu Children	—	0.15	0.02	0.45	—	5.4	0.9	4.0–6.5
	23-m/o Western Toddlers	0.67	0.38	0.88	0.093	1.9	0.3	1.5–2.0
	30-m/o Western Toddlers	0.50	0.25	0.75	0.780	2.5	0.3	2.2–2.9
	42-m/o Western Children	0.00	0.00	0.21	1.000	3.6	0.3	3.2–4.0
	60-m/o Western Children	0.00	0.00	0.21	1.000	5.4	0.3	5.0–5.8
Aka Adults	—	0.11	0.00	0.48	—	28.1	5.9	21.0–38.0
	23-m/o Western Toddlers	0.667	0.384	0.882	0.118	1.9	0.3	1.5–2.0
	30-m/o Western Toddlers	0.500	0.247	0.753	0.700	2.5	0.3	2.2–2.9
	42-m/o Western Children	0.000	0.000	0.206	1.000	3.6	0.3	3.2–4.0
	60-m/o Western Children	0.000	0.000	0.206	1.000	5.4	0.3	5.0–5.8

Confidence limits of the proportions of groups using emulation were calculated using the Clopper-Pearson (exact) method. For statistical tests, Fisher’s exact test and the Holm-Bonferroni method were used to obtain p-values corrected for multiple comparisons. Ages are given in years as precision beyond half a year was not known for Aka and Ngandu participants. Data from Western toddlers and children in the United Kingdom were obtained from McGuigan et al. [26] and McGuigan and Whiten [22]. Western toddlers and children were classified by their dominant approach, as in the source texts.

### Task Efficiency

The length of time each participant spent interacting with the apparatus was significantly predicted by the number of irrelevant actions performed (*β* = 3.984, t(34) = 3.805, p < .001), by irrelevancy quotients (*β* = 27.654, t(34) = 3.564, p = .001), and by decreasing use of an emulative social learning mechanism (*β* = −16.625, t(34) = −3.186, p = .003), but not by irrelevant imitation scores or fidelity quotients. The number of irrelevant actions, irrelevancy quotients, and social learning mechanism were found to explain a significant proportion of the variance in test duration (number: r^2^ = .278, F(1, 34) = 14.48, p < .001; irrelevancy: r^2^ = .251, F(1, 34) = 12.70, p = .001; mechanism: r^2^ = .207, F(1, 34) = 10.15, p = .003), while irrelevant imitation scores and fidelity quotients did not. The participant’s sex was found to have no effect on test duration and no significant interactions were found. Participant age was a significant predictor of test duration among Aka children and Ngandu children, with older ages associated with longer times (*β* = 7.443, t(25) = 2.176, p = .039) and with age explaining a significant proportion of the variance in test duration (r^2^ = .126, F(1, 25) = 4.736, p = .039), but no difference was found between these groups and there was no significant interaction between age and group.

## Discussion

In order to infer a difference between groups in the tendency to overimitate, it must first be determined at what rates the demonstrated irrelevant actions would be produced in the absence of a demonstration and whether these rates differ. Bias could exist if the actions were somehow intuitive to the task or were themselves affected by cultural, developmental, or environmental factors. Our results suggest that this is not the case (see **Fig. 1**). Participants that received a demonstration displayed a greater number of irrelevant actions than those that did not receive a demonstration, indicating that some degree of social learning took place. That there were no significant differences between groups within the no demonstration condition suggests that such differences either do not exist or were undetectable due to the small sample of individuals that chose to engage with the box in the no demonstration condition. Notably, few of the irrelevant actions observed in the no demonstration condition matched those modeled in the demonstration. This suggests that participants that did not receive a demonstration were merely trying to determine the function of the box through exploration or play. These results indicate that the irrelevant actions exhibited by participants exposed to a demonstration were copied from that demonstration by means of imitation rather than the result of individual learning or specific biases.

In comparing the reproduction of irrelevant actions between Aka children and Aka adults, children copied fewer irrelevant actions (see **Fig. 2**), and had lower irrelevant imitation scores (see **Fig. 3**). Though Ngandu children were intermediate between Aka children and Aka adults in all three measures of irrelevancy, they were only statistically distinguishable from Aka children, and only in irrelevant imitation scores rather than the number of irrelevant actions. This may suggest that a proportional measure is more informative than the raw count data. Although the irrelevancy quotient has the advantage of being comparable across studies with differing methodologies and was seen to be a significant predictor of the time to reward retrieval, the small sample sizes of this study restricted its statistical power. Considering all three measures, Aka children were found to copy the demonstrated irrelevant actions at lower rates than both Aka adults and Ngandu children.

In accordance with the results found for irrelevant actions performed, Aka children were seen to have lower fidelity to the demonstrated sequence than Aka adults (see **Fig. 3**). Ngandu children were again intermediate to both groups but only statistically distinguishable from Aka adults. As overimitation involves a high degree of fidelity to the demonstrated sequence as well as the production of irrelevant actions, these two sources of data together suggest that Aka children were less likely to engage in overimitation than both Aka adults and Ngandu children.

When examined strictly on the basis of the social learning mechanism used (see **Fig. 4**), this trend becomes much more pronounced: 60% of Aka children that succeeded in the task did so by using emulation rather than imitation, compared to 15% of Ngandu children and 11% of Aka adults. Therefore, not only were Aka children engaging in overimitation much less frequently than the closely associated but culturally distinct Ngandu, they were also overimitating less than the adults in their own community. Comparisons with toddlers and children from the United Kingdom (see **Fig. 4; Table 1**) reveal that Aka children also displayed patterns of social learning distinct from those being used by their developmental peers in a typical Western culture. In previous Western studies focusing on early to middle childhood, the frequency of the use of overimitation has been seen to increase with age while the use of emulation decreases [20, 21, 25–27], such that Western children of comparable age to the Aka tested here overimitate almost exclusively, thereby utilizing a non-selective copying strategy which has been shown to be less efficient at accomplishing complex tasks in the short term ([22, 26] and present study). Aka children, by contrast, seem to readily filter out this costly irrelevant information, much as Western infants and toddlers do [22, 23].

A number of explanations could ultimately be responsible for the observed cross-cultural differences in childhood overimitation. Firstly, there are fundamental differences between hunter-gatherer societies such as the Aka and more Westernized populations. These differences are exemplified by three foundational schemas among the Aka, which are also common among other hunter-gatherer groups: *egalitarianism*, or equal respect for all individuals; *autonomy*, or the ability of each individual to do as he or she wishes; and *sharing*, that resources and responsibilities are spread between members of the group (see **S2 Appendix** and [4] for more information and for foundational schemas among the Ngandu). The cultural beliefs represented by foundational schemas shape people’s perspectives on their social relationships, interactions, and values, as well as their perception of and interactions with the world around them. Culture can thus dictate the ways in which people learn by influencing whom they pay attention to, which aspects of a model’s behavior are most salient to them, and how they perceive and approach a novel problem. These features of individual and social learning are commonly investigated through the action of cognitive biases, including frequency-dependent biases such as conformity and model-based biases of individual aspects like prestige [9, 34, 60].

In any experimental study, one must consider the importance of the testing environment and its relevance to the participant’s culturally normative experience. In this experiment, participants were tested in naturalistic settings and demonstrations were performed by an ingroup demonstrator. Experiments conducted in an artificial laboratory space, with video demonstrations rather than live ones, or by demonstrators of a different gender, age, or ethnic or socioeconomic group relative to the observer could find results that contrast with those of studies that take these factors into account [60, 61]. These effects could be even more pronounced in small-scale cultures where these kinds of experiences and interactions would be less familiar. Nielsen and Tomaselli [27] did use ingroup models for some tests among the !Xun and Khwe San and failed to find a significant difference between Western and local models, though this shared community has a complex intercultural history with significant Western influences (see **S1 Appendix**). An important observation is that rates of overimitation are lower in Western studies in which the demonstrator is the same age or younger than the observer [28, 62–64]. This is likely due to the perception of an older model as an individual of greater status and experience that expects deference and respect, a cultural value common in Western groups but rare in small-scale hunter-gatherers. The egalitarian nature of Aka society leads to the perception of other Aka as equal regardless of age or status but does call for respect for members of outgroups with greater influence such as the Ngandu. For this study, initially an adult Ngandu man was trained to model for the Aka participants, but many Aka children—particularly girls—declined to participate out of deference to him. Thus, the identity of the demonstrator and the participants’ cultural views on equality and status can be very important components of an experiment. The testing environment should also be carefully considered in research on non-human primates and other animals, particularly when experiments are performed under conditions that differ starkly from the natural ecology of the species or when actions are demonstrated by humans rather than an intraspecific model.

Differences in levels of overimitation could also be driven by behavioral responses to culturally constructed environmental niches [65]. Aka children may typically be presented with objects that are causally transparent (or at least translucent) rather than causally opaque. This contrasts with the Western case in which so many of our modern technological artifacts, including the toys and other objects that our children are exposed to from an early age, have visible effects that are connected only in a very loose sense to the actions performed upon them. This scenario could lead to blanket copying via overimitation being an advantageous strategy for young children in a Western context but not in a small-scale non-Western one in which emulation could be the more efficient approach. Overimitation could still be expected to increase with age in both settings as the complexity of skills being acquired increases. In small-scale cultures, the number of particular tools and social rituals with causally opaque functions tends to increase with age, so the transmission and acquisition of these skills could be aided by overimitation at older ages. However, this explanation is unlikely to apply uniformly to the wide diversity of practices among non-Western cultures.

The final—and potentially most powerful—explanation presented for the cultural differences observed here is that Western attitudes on and approaches to education fundamentally structure how children “learn to learn” from an early age. Even before children are enrolled in formal schooling, parents begin the pedagogical process by applying the same cultural ideas and practices regarding teaching that were used in their own education as, in a modern Western context, the act of parenting is itself equated with teaching [66]. Though so-called “Western” schooling has a high degree of variation and has been introduced to the world’s non-Western societies at different times by different educational traditions [67], it is in all cases markedly different from the environmental context of social learning in hunter-gatherers and other small-scale groups. Aka children learn the majority of their skills through observation, imitation, and participation. Some teaching—defined as the modification of one’s behavior to facilitate learning of information, knowledge, or skills in another—does occur among the Aka, but formal teaching as experienced in a classroom by Western children is absent [4, 68]. The premise that learning is itself culturally learned is supported by a recent study by Mesoudi et al. [69], which found cross-cultural variation in the use of social learning strategies between a group of mainland Chinese students and other dispersed groups with varying levels of Western influence.

In non-Western societies, the introduction of Western schooling has marked effects on the value placed on traditional skills, knowledge, and attitudes and on the transmission of these cultural variants, and often serves to integrate peripheral groups into dependent and less-privileged roles in the dominant culture [70–73]. Nielsen et al. [40] note that the groups of San and Aboriginal Australians tested in the only non-Western studies of overimitation all had access to formal schooling, albeit at different levels and with opportunities most limited among the ‡Khomani San. The Ngandu of Bagandu have had access to primary school for at least 40 years, meaning that most Ngandu adults have had some level of formal education and that younger children have older siblings and peers that regularly attend school. The Aka in the study area have had only limited access to local services and, consequently, none of the Aka children or Aka adults that participated in this study have ever attended school (see **S1 Appendix**). The potential influence of these ontogenetic and cultural effects on the dynamic processes of social learning and cognition should not be understated.

## Conclusions

This study has demonstrated that cross-cultural variation exists in the degree to which overimitation is expressed at particular ages in human children. Our data indicate that pronounced overimitation does not universally emerge in early childhood; specifically, overimitation may emerge later in life within small-scale, non-Western cultures. In contrast to Aka children, who were found to be distinctly emulative, Aka adults displayed a high degree of overimitation comparable to levels seen in Western populations. Ngandu children, with greater access to Western influences and formal education, as well as cultural views and values that contrast sharply with the Aka, also more closely resembled Western groups in their tendency to overimitate. As a feature of social learning with possible implications for biological and cultural evolution, overimitation merits further investigation across cultural, socioeconomic, and non-human groups to gain a better understanding of its cognitive underpinnings and influences from cultural, ontogenetic, and ecological factors.

There are notable limitations to the present study that point toward future directions in research. The ongoing political crisis in the Central African Republic prevented the enlargement of our sample size and the inclusion of Ngandu adults, which would allow for improved statistical power and a more balanced assessment of overimitation across age groups in both cultures. A shortfall of this study and others is the lack of attention on middle to late childhood and adolescence which could shed light on changes in social learning strategies between developmental periods across the lifespan [74]. Future study on infants and toddlers in small-scale groups could also track the development of overimitation at earlier ages than those examined here. This study did compare overimitation in both children and adults among the Aka, which has not been done for any other non-Western population and could provide insight into the developmental components of overimitation.

The primary intended significance of this work is to echo recent introspection in the behavioral, cognitive, social, and cultural sciences by Arnett [14], Henrich et al. [3], and others urging the importance of avoiding sampling biases, even when cross-cultural sampling efforts can be difficult or expensive. Culture’s effects on human behavior are varied and profound and not easily discerned from a subsample of one’s own cultural group. This paper describes one of the few contributions toward the cross-cultural study of childhood social learning and overimitation. Though we believe the Aka at this field site are more representative of small-scale, non-Western societies than those previously studied due to their limited Western influences and continued dependence upon hunting and gathering for their daily subsistence, it is also clear that no two cultures are identical, whether they be Western or non-Western in nature. For perspective, one must consider the range of cultural differences among the many groups of Aka and other forest foragers of the Congo Basin, which number over 350,000 people and live in a variety of conditions [53, 75]. The Aka studied here are but one example of the diversity of ancient and modern small-scale cultures that have inhabited our planet.

## Supporting Information

S1 Appendix Contrasting Western influences on the San, Aka, and Ngandu. Additional comments on the Western cultural influences on the San populations studied by Nielsen and Tomaselli [27] and Nielsen et al. [40] and on the Aka and Ngandu of the present study.
(DOC)

S2 Appendix Foundational schemas of the Aka and Ngandu. Detailed descriptions of the culturally distinct ways of thinking that structure many aspects of life for the Aka and Ngandu.
(DOC)

S3 Appendix Methods and statistical analyses. Notes on methodology, statistical procedures performed, and the reproduction of figures.
(DOC)

S1 Table Type II ANOVA tables from Control analyses. Includes ANOVA tables, partial eta squared values, confidence intervals, and results of Tukey’s HSD post-hoc tests.
(DOC)

S2 Table Type II ANOVA tables from Reproduction of Irrelevant Actions analyses. Includes ANOVA tables, ANCOVA test result for age effects, partial eta squared values, confidence intervals, and results of Tukey’s HSD post-hoc tests.
(DOC)

S3 Table Type II ANOVA table from Copying Fidelity analysis. Includes ANOVA table, ANCOVA test result for age effects, partial eta squared values, confidence intervals, and results of Tukey’s HSD post-hoc tests.
(DOC)

S1 Dataset Overimitation in the Aka and Ngandu. Full dataset used in analyses and the generation of figures, in CSV format. Contains information on participant group, age, sex, experimental setting, demonstrations, success, tool and door use, irrelevant actions, calculated measures of irrelevancy and fidelity, test duration, and social learning mechanism used. Age is in years and test duration is in seconds. See text for description of variables.
(CSV)
