# Supplementary material for: Cultural Variation in the Use of Overimitation by the Aka and Ngandu of the Congo Basin
Source: PLoS One. 2015 Mar 27;10(3):e0120180. doi: 10.1371/journal.pone.0120180 (PMC4376636; doi:10.1371/journal.pone.0120180)
Supplement: S1 Appendix — Additional comments on the Western cultural influences on the San populations studied by Nielsen and Tomaselli [27] and Nielsen et al. [40] and on the Aka and Ngandu of the present study. (DOC) [file pone.0120180.s001.doc]

**Cultural variation in the use of overimitation by the Aka and Ngandu of the Congo Basin: Supporting information**

Richard E.W. Berl^1*^, Barry S. Hewlett^2^

^1^ School of Biological Sciences, Washington State University, Pullman, Washington, United States of America

^2^ Department of Anthropology, Washington State University Vancouver, Vancouver, Washington, United States of America

* Corresponding author

E-mail: richard.berl@wsu.edu (REWB)

# S1 Appendix: Contrasting Western influences on the San, Aka, and Ngandu

The San groups studied by [Nielsen and Tomaselli [1]](#_ENREF_1) all resettled in their present locations as a result of government land grants and purchases in the late 1990s and early 2000s , aside from the mixture of !Xóõ San and Bakgalagadi Bantu living in Botswana that were forced to the periphery of their territory and have only recently regained limited land rights . The ǂKhomani adjacent to the Kgalagadi Transfrontier Park spanning the South Africa-Botswana border moved into the area more than a quarter century after all indigenous groups were forcibly ejected in 1973, while the !Xun and Khwe, originally from Angola, arrived in the vicinity of Kimberley after fleeing war in Namibia. The !Xun and Khwe have no historical ties with each other or with the ancestral San populations of the land which they now inhabit . Before taking up residence in these areas, many of the ǂKhomani had been working as rural farm workers and laborers or in cultural tourism and the !Xun and Khwe migrants had been subject to military service . [Nielsen et al. [10]](#_ENREF_10) provide additional information on these San populations, which were the focus of the study by [Nielsen and Tomaselli [1]](#_ENREF_1), recognizing that issues such as poverty, disease, malnutrition, unemployment, alcoholism, and exploitation have arisen in these communities as a result of civil and international war, apartheid policies, and other major Western influences. This contrasts with the initially more simplified representation of the San as “recent descendents of true hunter-gatherers living in communities where many aspects of traditional culture are maintained” and more accurately reflects the long history of Western influences on San cultures. For these reasons, Robins describes the San as being neither “indigenous people untouched by modernity,” nor “modern citizens completely moulded by discourses of western democracy and liberal individualism,” rather an amalgam of the two distinct cultures with their own beliefs and values.

Among the !Xun and Khwe, 18% of adults have had no formal education . For context, 11.3% of primary school-aged children across South Africa are out of school, many of which are expected to never attend school . Therefore, the level of schooling among these San groups is not exceptionally low and may be expected to increase in the current generation due to the presence of a primary and secondary school in their shared settlement.

The village of Bagandu in which the Ngandu live is a small rural settlement in which people subsist on an annual household income of roughly 500 USD obtained through the sale of cash crops. Malnutrition is rare, but infant and child mortality rates are high due to infectious and parasitic diseases as in most small-scale cultures . Few salaried governmental positions exist and almost everyone in the village is heavily involved in subsistence farming, foraging, or a mixture of the two. Catholic missionaries have been in the village for roughly 20 years and have established a small hospital. Logging companies operate within 50 km of the village and trucks drive through town. The village is not served by electricity or running water and rough dirt roads are unmaintained and often inaccessible.

The Ngandu of Bagandu have had access to primary school for at least 40 years, meaning that most Ngandu adults have had some level of formal education. Ngandu children that qualify may go on to secondary school in Mbaiki and Bangui, 60 and 165 km away, respectively. The Ngandu children that participated in this study had not yet begun schooling but many would do so soon, had older siblings attending, and had parents with some formal education.

Researchers in the study area have organized sporadic instruction for Aka on some basic skills—counting, reading, and writing in French—on an informal basis since 2003 but not for the past several years. To our knowledge, this constitutes the only exposure to Western-style instruction among the Aka within the study area. Aka in other areas of the Central African Republic have been provided with primary education through Catholic missionaries since the mid-1990s. Patterns of Western cultural interaction vary widely among other forest forager groups beyond the boundaries of the study area . In contrast with many other small-scale cultures, including the San of South Africa and Botswana that have been the focus of previous overimitation research, the Aka of this study live under conditions of relative isolation from Western influence.

# References

1. Nielsen M, Tomaselli K. Overimitation in Kalahari Bushman children and the origins of human cultural cognition. Psychol Sci. 2010;21(5):729-36.

2. Pamo B. San language development for education in South Africa: The South African San Institute and the San language committees. Diaspora, Indigenous, and Minority Education. 2011;5(2):112-8.

3. Tomaselli KG. Where global contradictions are sharpest: Research stories from the Kalahari. Amsterdam: Rozenberg Publishers; 2005.

4. Hitchcock RK, Biesele M, Lee RB. The San of Southern Africa: A status report, 2003. Arlington, VA: American Anthropological Association, 2003.

5. den Hertog TN. Diversity behind constructed unity: The resettlement process of the !Xun and Khwe communities in South Africa. J Contemp Afr Stud. 2013;31(3):345-60.

6. Hitchcock RK. 'We are the First People': Land, natural resources and identity in the Central Kalahari, Botswana. J S Afr Stud. 2002;28(4):797-824.

7. Taylor JJ. Celebrating San victory too soon? Reflections on the outcome of the Central Kalahari Game Reserve case. Anthropol Today. 2007;23(5):3-5.

8. Robins S. NGOs, 'Bushmen' and double vision: The ≠khomani San land claim and the cultural politics of 'community' and 'development' in the Kalahari. J S Afr Stud. 2001;27(4):833-53.

9. Robins S, Madzudzo E, Brenzinger M. An assessment of the status of the San in South Africa, Angola, Zambia and Zimbabwe. Windhoek: Legal Assistance Centre, 2001 Contract No.: 2.

10. Nielsen M, Mushin I, Tomaselli K, Whiten A. Where culture takes hold: ‘Overimitation’ and its flexible deployment in Western, Aboriginal and Bushmen children. Child Dev. 2014;85(6):2169–84.

11. UNESCO Institute for Statistics, UNICEF. Global initiative on out-of-school children: Eastern and Southern Africa region. Montreal & New York: UNESCO Institute for Statistics & UNICEF, 2013.

12. Hewlett BS. Demography and childcare in preindustrial societies. J Anthropol Res. 1991;47(1):1-37.

13. Hewlett BS. Cultural diversity among African pygmies. In: Kent S, editor. Cultural diversity among twentieth-century foragers: An African perspective: Cambridge University Press; 1996. p. 215-24.

14. Bahuchet S. Cultural diversity of African Pygmies. In: Hewlett BS, editor. Hunter-gatherers of the Congo Basin: Cultures, histories, and biology of African Pygmies. New Brunswick, NJ: Transaction Publishers; 2014. p. 1-30.
