# Supplementary material for: Cultural Variation in the Use of Overimitation by the Aka and Ngandu of the Congo Basin
Source: PLoS One. 2015 Mar 27;10(3):e0120180. doi: 10.1371/journal.pone.0120180 (PMC4376636; doi:10.1371/journal.pone.0120180)
Supplement: S2 Appendix — Detailed descriptions of the culturally distinct ways of thinking that structure many aspects of life for the Aka and Ngandu. (DOC) [file pone.0120180.s002.doc]

**Cultural variation in the use of overimitation by the Aka and Ngandu of the Congo Basin: Supporting information**

Richard E.W. Berl^1*^, Barry S. Hewlett^2^

^1^ School of Biological Sciences, Washington State University, Pullman, Washington, United States of America

^2^ Department of Anthropology, Washington State University Vancouver, Vancouver, Washington, United States of America

* Corresponding author

E-mail: richard.berl@wsu.edu (REWB)

# S2 Appendix: Foundational schemas of the Aka and Ngandu

Three related foundational schemas—or, values and ways of thinking that pervade many domains of life—define Aka culture and that of many other hunter-gatherers: egalitarianism, autonomy, and sharing . *Egalitarianism* represents the belief that others are respected for what they are and that it is inappropriate to draw attention to oneself or to judge others as better or worse. Men and women of all ages are viewed as relatively equal and have similar access to resources. Respect for individual *autonomy* is also a core value. One does not coerce or tell others what to do, including children. Each individual does more or less what he or she wants: if a man does not feel like hunting one day, he does not do so; if an infant attempts to play with a machete, she is allowed to do so. A giving or *sharing* attitude also structures hunter-gatherer life. The Aka share 50-80% of what is acquired through hunting and gathering, they share it with everyone in camp, and they share it every day . Sharing of childcare is also extensive; for instance, 90% of Aka mothers reported that other women nursed their young babies .

Foundational schemas among the Ngandu include: gender and age hierarchy, communalism, and material or economic dimensions of social relations. Ngandu believe that women should defer to the requests of men and that the young should be respectful of and listen to those older than them, be they older siblings or parents. *Hierarchies* are enforced through patrilocality, patrilineality, and strong clan organization. *Communalism* refers to a value placed on the needs of the group—generally clan members or extended family—over the needs of an individual. The *material or economic dimensions of social relationships* mean that interpersonal relations should have economic or material components above and beyond purely social and emotional aspects. Merely liking a person or being kind is not enough to sustain a relationship. This contrasts with Aka relationships, which place a greater emphasis on social and emotional bonds.

Foundational schemas play an influential role in structuring the social life and cultural traits of human groups. It is also likely that they have important effects on the dynamics of social learning and how members of different cultures “learn to learn” (see Conclusions in text). For these reasons, differing foundational schemas among the Aka, Ngandu, and other Western and non-Western groups are important considerations in the investigation of cultural influences on social and cognitive phenomena.

# References

1. Hewlett BS, Fouts HN, Boyette AH, Hewlett BL. Social learning among Congo Basin hunter-gatherers. Philos Trans R Soc B. 2011;366:1168-78.

2. Shore B. Culture in mind: Cognition, culture, and the problem of meaning: Oxford University Press; 1996.

3. Kitanishi K. Food sharing among the Aka hunter-gatherers in northeastern Congo. Afr Stud Monogr. 1998;25(Supplementary Issue):3-32.

4. Hewlett BS, Winn S. Allomaternal Nursing in Humans. Curr Anthropol. 2014;55:200-29.
