# Supplementary material for: Cultural Variation in the Use of Overimitation by the Aka and Ngandu of the Congo Basin
Source: PLoS One. 2015 Mar 27;10(3):e0120180. doi: 10.1371/journal.pone.0120180 (PMC4376636; doi:10.1371/journal.pone.0120180)
Supplement: S3 Appendix — Notes on methodology, statistical procedures performed, and the reproduction of figures. (DOC) [file pone.0120180.s003.doc]

**Cultural variation in the use of overimitation by the Aka and Ngandu of the Congo Basin: Supporting information**

Richard E.W. Berl^1*^, Barry S. Hewlett^2^

^1^ School of Biological Sciences, Washington State University, Pullman, Washington, United States of America

^2^ Department of Anthropology, Washington State University Vancouver, Vancouver, Washington, United States of America

* Corresponding author

E-mail: richard.berl@wsu.edu (REWB)

# S3 Appendix: Methods and statistical analyses

Due to demonstrator error, some participants received an irregular number of demonstrations: two Aka children received four demonstrations (participants #1 and #3), one Aka child received five (#2), one Ngandu child received two (#14), and one Aka adult received four (#1). Given the small sample sizes available for this study, these participants were retained in the data set and their inclusion was not found to cause any directional bias in results. Two participants, one Ngandu child (#26) and one Aka adult (#4), were excluded as outliers due to the number of irrelevant actions they performed, which were greater than two standard deviations above the mean and were inflating the significance of statistical relationships. Neither of these participants received an irregular number of demonstrations. Participation rates in engaging with the task were roughly equal between groups (Fisher’s Exact, p = .65).

Whether the tool was or was not used to perform each action was noted for each participant, as well as the method used to open the relevant front door of the box. In the demonstration, the front door was slid to the right but a number of participants in the demonstration condition slid the door to the left (Aka children: *N* = 2; Ngandu children: *N* = 4; Aka adults: *N* = 3). One participant, an Ngandu child, discovered the alternate method of lifting the front door to gain access to the box. All relationships involving these two factors were found to be nonsignificant and there were no significant differences by group in use of the tool or door opening method, so neither were included in the overall models.

Data were analyzed using three-way ANOVA tests to examine differences in the number of irrelevant actions, irrelevant imitation scores, irrelevancy quotients, and fidelity quotients by demonstration condition, group, and sex. A multivariate test was not used because the first three measures could be seen as equivalent. For subsequent tests on these measures looking only at participants in the demonstration condition, two-way ANOVA tests were used with group and sex as factors. As data were unbalanced, Type III sums of squares were used and, if no significant interaction term was found, results from Type II sums of squares were provided. ANCOVA tests to examine the effects of participant age and group on calculated measures excluded Aka adults to avoid confounding group with age. Tukey’s honest significant difference tests were used for multiple (post-hoc) comparisons and Welch’s t-tests and Pearson’s Chi-squared tests were performed in some cases to ease comparison of results with previous studies. Fisher’s exact test and linear regression were used to examine the effects of social learning mechanism and effects of measures on test duration. In comparisons with data on social learning mechanisms from previous Western studies, Holm-Bonferroni corrections were used to adjust p-values for multiple comparisons and the Clopper-Pearson (exact) method was used to calculate confidence intervals. Analyses were performed using the R statistical package, version 3.1.0 . p-values of .05 or lower were considered to be statistically significant.

In order to replicate results obtained here and displayed in figures, the following individuals must be excluded from the data in **S1 Dataset** of the supplementary information: Aka adult #4 for displaying 17 irrelevant actions by alternately sliding and tapping the top of the box repeatedly, Ngandu child #16 (already removed by authors) for “cheating” by watching another individual’s test before his own, and Ngandu child #26 for displaying 14 irrelevant actions by alternately tapping the sides of the box a number of times. The two removed for high numbers of irrelevant actions were excluded as outliers for reasons mentioned above. Full ANOVA tables of results are available as supporting information **S1 Table**, **S2 Table**, and **S3 Table**.

# References
