# Supplementary material for: Cultural Variation in the Use of Overimitation by the Aka and Ngandu of the Congo Basin
Source: PLoS One. 2015 Mar 27;10(3):e0120180. doi: 10.1371/journal.pone.0120180 (PMC4376636; doi:10.1371/journal.pone.0120180)
Supplement: S1 Table — Includes ANOVA tables, partial eta squared values, confidence intervals, and results of Tukey’s HSD post-hoc tests. (DOC) [file pone.0120180.s004.doc]

**Cultural variation in the use of overimitation by the Aka and Ngandu of the Congo Basin: Supporting information**

Richard E.W. Berl^1*^, Barry S. Hewlett^2^

^1^ School of Biological Sciences, Washington State University, Pullman, Washington, United States of America

^2^ Department of Anthropology, Washington State University Vancouver, Vancouver, Washington, United States of America

* Corresponding author

E-mail: richard.berl@wsu.edu (REWB)

**S1 Table.** Type II ANOVA tables from Control analyses.

| **Response** | **Factor** | **F** | **d.f.** | **p** | **ƞ_p_^2^** | **Lower 95% CI** | | **Upper 95% CI** | | **Tukey's HSD Adjusted p** |
| --- | --- | --- | --- | --- | --- | --- | --- | --- | --- | --- |
| Number of Irrelevant Actions | Condition | 4.138 | 1, 45 | **0.048** | 0.084 | 0.275 | | 2.387 | | **0.016** |
|  | Group | 3.709 | 2, 45 | **0.032** | 0.142 | AC-NC | -0.474 | AC-NC | 2.505 | 0.235 |
|  |  |  |  |  |  | AC-AA | 0.232 | AC-AA | 3.929 | **0.024** |
|  |  |  |  |  |  | NC-AA | -0.723 | NC-AA | 2.852 | 0.328 |
|  | Sex | 2.306 | 1, 45 | 0.136 | 0.049 | -0.285 | | 2.034 | | 0.155 |
| Irrelevant Imitation Score | Condition | 0.158 | 1, 40 | 0.693 | 0.004 | -0.120 | | 0.233 | | 0.660 |
|  | Group | 4.858 | 2, 40 | **0.013** | 0.195 | AC-NC | 0.072 | AC-NC | 2.505 | **0.009** |
|  |  |  |  |  |  | AC-AA | -0.019 | AC-AA | 0.578 | 0.071 |
|  |  |  |  |  |  | NC-AA | -0.342 | NC-AA | 0.250 | 0.924 |
|  | Sex | 0.411 | 1, 40 | 0.525 | 0.010 | -0.133 | | 0.256 | | 0.545 |
| Irrelevancy Quotient | Condition | 1.462 | 1, 45 | 0.233 | 0.031 | -0.441 | | 0.110 | | 0.308 |
|  | Group | 3.597 | 2, 45 | **0.036** | 0.138 | AC-NC | 0.023 | AC-NC | 0.562 | **0.030** |
|  |  |  |  |  |  | AC-AA | -0.065 | AC-AA | 0.604 | 0.136 |
|  |  |  |  |  |  | NC-AA | -0.347 | NC-AA | 0.300 | 0.983 |
|  | Sex | 0.354 | 1, 45 | 0.555 | 0.008 | -0.148 | | 0.272 | | 0.574 |
| Fidelity Quotient | Condition | 18.822 | 1, 51 | **<< .001** | 0.270 | 0.198 | | 0.539 | | **<< .001** |
|  | Group | 5.201 | 2, 51 | **0.009** | 0.169 | AC-NC | -0.092 | AC-NC | 0.293 | 0.426 |
|  |  |  |  |  |  | AC-AA | 0.076 | AC-AA | 0.535 | **0.006** |
|  |  |  |  |  |  | NC-AA | -0.024 | NC-AA | 0.435 | 0.088 |
|  | Sex | 0.023 | 1, 51 | 0.879 | < .001 | -0.133 | | 0.155 | | 0.881 |

Confidence intervals from comparisons between groups were obtained from Tukey's HSD tests.
