# Supplementary material for: Cultural Variation in the Use of Overimitation by the Aka and Ngandu of the Congo Basin
Source: PLoS One. 2015 Mar 27;10(3):e0120180. doi: 10.1371/journal.pone.0120180 (PMC4376636; doi:10.1371/journal.pone.0120180)
Supplement: S2 Table — Includes ANOVA tables, ANCOVA test result for age effects, partial eta squared values, confidence intervals, and results of Tukey’s HSD post-hoc tests. (DOC) [file pone.0120180.s005.doc]

**Cultural variation in the use of overimitation by the Aka and Ngandu of the Congo Basin: Supporting information**

Richard E.W. Berl^1*^, Barry S. Hewlett^2^

^1^ School of Biological Sciences, Washington State University, Pullman, Washington, United States of America

^2^ Department of Anthropology, Washington State University Vancouver, Vancouver, Washington, United States of America

* Corresponding author

E-mail: richard.berl@wsu.edu (REWB)

**S2 Table.** Type II ANOVA tables from Reproduction of Irrelevant Actions analyses.

| **Response** | **Factor** | **F** | **d.f.** | **p** | **ƞ_p_^2^** | **Lower 95% CI** | | **Upper 95% CI** | | **Tukey's HSD Adjusted p** |
| --- | --- | --- | --- | --- | --- | --- | --- | --- | --- | --- |
| Number of Irrelevant Actions | Group | 3.706 | 2, 37 | **0.034** | 0.167 | AC-NC | -0.819 | AC-NC | 2.694 | 0.402 |
|  |  |  |  |  |  | AC-AA | 0.243 | AC-AA | 4.382 | **0.026** |
|  |  |  |  |  |  | NC-AA | -0.695 | NC-AA | 3.445 | 0.249 |
|  | Sex | 1.948 | 1, 37 | 0.171 | 0.050 | 0.408 | | 2.211 | | 0.178 |
|  | Age (ANCOVA) | 1.584 | 1, 29 | 0.197 | < .001 | -0.293 | | 1.232 | | — |
| Irrelevant Imitation Score | Group | 4.501 | 2, 35 | **0.018** | 0.205 | AC-NC | 0.017 | AC-NC | 0.516 | **0.034** |
|  |  |  |  |  |  | AC-AA | 0.019 | AC-AA | 0.596 | **0.035** |
|  |  |  |  |  |  | NC-AA | -0.248 | NC-AA | 0.329 | 0.936 |
|  | Sex | 0.619 | 1, 35 | 0.437 | 0.017 | -0.114 | | 0.258 | | 0.446 |
|  | Age (ANCOVA) | 0.145 | 1, 27 | 0.706 | 0.007 | -0.133 | | 0.091 | | — |
| Irrelevancy Quotient | Group | 3.332 | 2, 37 | **0.036** | 0.153 | AC-NC | -0.022 | AC-NC | 0.528 | 0.077 |
|  |  |  |  |  |  | AC-AA | -0.027 | AC-AA | 0.622 | 0.078 |
|  |  |  |  |  |  | NC-AA | -0.280 | NC-AA | 0.369 | 0.940 |
|  | Sex | 0.372 | 1, 37 | 0.546 | 0.010 | -0.143 | | 0.267 | | 0.552 |
|  | Age (ANCOVA) | 0.062 | 1, 29 | 0.802 | 0.006 | -0.113 | | 0.145 | | — |

ANCOVA results are presented for the main effect of age only and also included group (Aka children or Ngandu children) as a factor. Confidence intervals from comparisons between groups were obtained from Tukey's HSD tests.
