# Supplementary material for: Cultural Variation in the Use of Overimitation by the Aka and Ngandu of the Congo Basin
Source: PLoS One. 2015 Mar 27;10(3):e0120180. doi: 10.1371/journal.pone.0120180 (PMC4376636; doi:10.1371/journal.pone.0120180)
Supplement: S3 Table — Includes ANOVA table, ANCOVA test result for age effects, partial eta squared values, confidence intervals, and results of Tukey’s HSD post-hoc tests. (DOC) [file pone.0120180.s006.doc]

**Cultural variation in the use of overimitation by the Aka and Ngandu of the Congo Basin: Supporting information**

Richard E.W. Berl^1*^, Barry S. Hewlett^2^

^1^ School of Biological Sciences, Washington State University, Pullman, Washington, United States of America

^2^ Department of Anthropology, Washington State University Vancouver, Vancouver, Washington, United States of America

* Corresponding author

E-mail: richard.berl@wsu.edu (REWB)

**S3 Table.** Type II ANOVA tables from Copying Fidelity analyses.

| **Response** | **Factor** | **F** | **d.f.** | **p** | **ƞ_p_^2^** | **Lower 95% CI** | | **Upper 95% CI** | | **Tukey's HSD Adjusted p** |
| --- | --- | --- | --- | --- | --- | --- | --- | --- | --- | --- |
| Fidelity Quotient | Group | 6.268 | 2, 39 | **0.004** | 0.243 | AC-NC | -0.134 | AC-NC | 0.342 | 0.541 |
|  |  |  |  |  |  | AC-AA | 0.124 | AC-AA | 0.690 | **0.003** |
|  |  |  |  |  |  | NC-AA | 0.014 | NC-AA | 0.592 | **0.038** |
|  | Sex | 0.128 | 1, 39 | 0.723 | 0.003 | -0.146 | | 0.209 | | 0.726 |
|  | Age (ANCOVA) | 0.005 | 1, 31 | 0.943 | 0.003 | -0.101 | | 0.109 | | — |

ANCOVA results are presented for the main effect of age only and also included group (Aka children or Ngandu children) as a factor. Confidence intervals from comparisons between groups were obtained from Tukey's HSD tests.
